# Phytogenic and Nutritional Strategies to Improve Milk Production and Microbiological Quality in Lactating Donkeys

**DOI:** 10.3390/ani15203060

**Published:** 2025-10-21

**Authors:** Ana-Maria Plotuna, Ionela Hotea, Ileana Nichita, Ionela Popa, Kalman Imre, Viorel Herman, Emil Tîrziu

**Affiliations:** Faculty of Veterinary Medicine, University of Life Sciences “King Mihai I” from Timisoara, 300645 Timișoara, Romania; anamaria.plotuna@usvt.ro (A.-M.P.); ileananichita@usvt.ro (I.N.); ionela.popa@usvt.ro (I.P.); kalmanimre@usvt.ro (K.I.); viorelherman@usvt.ro (V.H.); emiltirziu@usvt.ro (E.T.)

**Keywords:** donkey milk quality, nutrient digestibility, lactating jennies, phytogenic additive, sustainable donkey farming

## Abstract

Donkey milk is highly regarded for its nutritional, immunological and hypoallergenic properties. In this context, the global demand is increasing, and the challenges of low production and milk hygiene need to be addressed. This study evaluated the effects of dietary and phytogenic supplementation on milk yield, nutrient digestibility, and milk quality in lactating jennies (*Equus asinus*). All donkeys had unrestricted access to natural pasture during the study. In addition to grazing, animals were divided into three groups (n = 10 per group) that differed only in the type of supplemental feed. The control group (CG) received pasture grass with a corn-based supplement; Group 1 (G1) received the same basal feed enriched with sunflower meal and a phytogenic blend of medicinal plants; and Group 2 (G2) received the same compound feed as G1 but without the phytogenic additives. Over an eight-week period, milk production, apparent digestibility coefficients (dry matter, protein, fibre, and ether extract), and microbiological quality were assessed. G1 demonstrated the highest milk yield (*p* < 0.001), improved nutrient digestibility (e.g., crude protein digestibility: 57.89 ± 4.21%), and a significant reduction in total viable counts (TVC) from 2.848 ± 0.265 to 1.898 ± 0.404 log_10_ CFU/mL (*p* < 0.001), compared to CG and G2. The latter maintained relatively stable TVC values (2.930 ± 0.260 → 2.838 ± 0.196; *p* = 0.356641), accompanied by reduced interindividual variability, whereas CG exhibited a slight increase (2.922 ± 0.253 → 2.949 ± 0.323; *p* = 0.792259) and greater variability, suggesting a negative trend. Crude protein digestibility was 55.86 ± 6.66% in G2 and 45.26 ± 9.85% in CG, further supporting the superior nutrient utilization efficiency observed in G1. The phytogenic supplement stabilized milk chemical composition, suggesting potential galactagogues, immunomodulatory, and antimicrobial effects. These findings support the use of functional feed additives as a promising strategy to enhance productive performance and milk hygiene in sustainable donkey farming systems.

## Simple Summary

Donkey milk is valued for its nutritional and hypoallergenic properties, but increasing milk yield and maintaining quality are challenges for farmers. This study tested how diets enriched with herbal supplements affect milk production, composition, and hygiene in lactating jennies. Over eight weeks, animals receiving the herbal supplement produced more milk, showed better nutrient use, and had lower bacterial counts in milk compared to the control group. These results suggest that phytogenic additives offer a natural and sustainable way to improve milk yield and quality in donkey farming.

## 1. Introduction

Over the past decade, donkey milk has re-emerged as a niche dairy product of significant nutritional and functional interest. It is characterized by a high-lactose, low-fat macro-profile and a protein composition that closely resembles human milk [1]. Recent studies report lactose concentrations ranging from 6.0 to 7.3 g per 100 mL, protein contents between 1.3 and 2.2 g per 100 mL, and fat levels as low as 0.1 to 1.4 g per 100 mL. Notably, 55 to 65% of the protein fraction consists of whey, resulting in a whey-to-casein ratio of approximately 1.5:1—significantly closer to that of human milk than to bovine milk [2,3]. In addition to these macronutrient similarities, donkey milk is rich in polyunsaturated fatty acids and bioactive proteins such as lysozyme, lactoferrin, and immunoglobulins, all of which contribute to its recognized antimicrobial and immunomodulatory properties [4,5,6]. This unique compositional profile underpins the growing appeal of donkey milk as both a functional food and a hypoallergenic alternative to conventional dairy sources [7,8,9,10]. Apart from its nutritional profile, donkey milk is gaining popularity as a hypoallergenic alternative for both infants and adults who are unable to tolerate bovine milk proteins [11]. Clinical observations suggest a significantly reduced IgE-mediated cross-reactivity in sensitive individuals [12]. This enhanced tolerability, combined with its naturally low fat content and minimal cholesterol levels, positions donkey milk as a unique and valuable dairy product in Europe and beyond [13,14,15].

However, the commercial production and use of donkey milk remains limited due to inherently low yields, <2.5 L per jenny per day. This low production is largely a result of the small alveolar-cisternal compartment and the necessity to milk shortly after separating the foal [16,17]. Consequently, research efforts have focused on nutritional and ethnoveterinary strategies aimed at safely stimulating lactation. Among these strategies, the use of plant-based supplements and herbal galactagogues has gained attention for their potential to enhance milk production effectively and naturally [18].

The growing interest in donkey milk has led to the emergence of specialized farms, where feeding management plays a pivotal role in sustaining lactation. Despite European initiatives to assess equine welfare, current criteria for adequate nutrition remain limited [19]. Research on the nutritional requirements of lactating jennies is insufficient, even though it is known that they efficiently utilize fibrous forages, even of poor quality, which are crucial for digestive health. At the same time, feeding diets enriched with concentrate feeds can improve milk quality by increasing nutrient content [19].

In this context, targeted nutritional strategies should include: balancing energy and protein intake to meet lactation demands, particularly in extensive systems where forage alone may be inadequate [20], incorporating oilseed by-products such as sunflower or soybean meal to increase protein availability, maintaining adequate fibre intake to support hindgut microbial fermentation and digestive function and introducing plant-derived compounds with known bioactive effects [21].

These phytogenic additives, including herbs, essential oils, and plant extracts, have demonstrated beneficial properties in various livestock species, such as stimulating digestive enzyme activity, modulating gut microbiota, and supporting metabolic and immune responses [22,23]. While donkey-specific studies are limited, broader findings indicate that these compounds may also improve milk yield, composition, and gastrointestinal health, offering promising perspectives for their inclusion in equine nutrition [22].

In Romania, National livestock data compiled by the Authority for Zootechnics registered 22.257 donkeys in 2022 [24]. While most animals are still kept in mixed smallholder systems, the past decade has seen the rise in specialized dairy enterprises, some milking 200–300 jennies under semi-intensive paddock management [25]. These developments underscore the need for evidence-based ration formulations that can raise output while safeguarding animal health and milk hygiene. In Romania, research on donkeys, particularly on feeding strategies and milk quality, remains limited, consisting of only a few descriptive studies on composition and hygienic status and isolated health reports, while controlled nutritional experiments are scarce [26,27,28].

The present study explores whether targeted herbal supplementation can enhance milk yield and hygienic quality in dairy donkeys raised under Romanian farm conditions, while monitoring fecal and forage-related indicators of animal health and energy balance. The findings aim to inform sustainable intensification of donkey dairying and contribute to the growing body of knowledge required for the sector’s safe expansion.

## 2. Materials and Methods

### 2.1. Ethics Statement

The study was approved by the Bioethics Committee under protocol number 201/09.03.2023 The research followed the guidelines of the Declaration of Helsinki and was conducted in accordance with the proper procedures of the University Veterinary Clinics, Faculty of Veterinary Medicine Timisoara and Romanian Veterinary College.

### 2.2. Experimental Design, Animals and Management

This study was conducted in Goruia, Caraș-Severin County, Romania, on a small-scale commercial donkey farm using an extensive management system, where animals grazed freely on pasture throughout the day. Milking was performed manually, as per the farm’s routine practice. The farm feeding management for all animals consists of pasture grass, consumed at an average of 10–11 kg/day, supplemented with approximately 1 kg of corn provided at morning milking (Table 1). The study was conducted during late-summer and early-autumn. A total of 30 lactating jennies were randomly selected from the herd and assigned into three groups (n = 10 per group). The jennies were of the Romanian local breed [29] (initial body weight 182 kg ± 10 kg with a BCS of 3) [30], closely matched in terms of physique and health status, with parities ranging from 2 to 6. The control group (CG) had an estimated 110 ± 2 days in milk (DIM), while both experimental groups, G1 and G2, had DIM values of 80 ± 6 days and 80 ± 4 days, respectively.

This research emphasizes the impact of concentrate and phytogenic supplementation on milk yield and quality in lactating donkeys, prioritizing the dietary interventions without detailed pasture characterization. The control group (CG) received no dietary intervention; animals in this group continued on their standard farm diet without any changes. This group was maintained as-is to preserve baseline conditions, and no supplements or alterations were introduced at any time. For the first experimental group (G1), a complete and balanced ration (Table 1) was formulated based on existing nutritional requirements and feeding recommendations for donkeys [19]. This ration included a phytogenic additive composed of a herbal blend (Table 2) known for its galactagogue properties [31,32]. The second experimental group (G2) received the same balanced ration as G1, formulated according to nutritional guidelines, but without the inclusion of phytogenic additives. The feeding protocol for the three groups was as follows:

- CG: 10–11 kg/animal/day of pasture grass supplemented with 1 kg of corn (Table 1);
- G1: 10–11 kg/animal/day of pasture grass supplemented with 1 kg/animal/day of compound feed including phytogenic additives (Table 1);
- G2: 10–11 kg/animal/day of pasture grass supplemented with 1 kg/animal/day of compound feed without phytogenic additives (Table 1).

The jennies were pre-adapted to the test feed for 2 weeks, after which they were fed the same feed daily for a period of 8 weeks.

The phytogenic additive (SC Hypericum Impex SRL, Baia Sprie, Maramureș, Romania) with the composition outlined in Table 2, was used with the intention of enhancing milk secretion. To guarantee uniformity and stability in the mixture, the additive was incorporated into the compound feed in pellet form. The compound pelleted feed was produced based on the ingredient composition detailed in Table 1 and Table 2, in three distinct batches to facilitate stringent quality control and consistency. The process involved grinding the raw materials, accurately dosing and homogenizing the ingredients, adding the binding agent (bentonite), and finally, pelleting, all in accordance with the European Feed Manufacturers’ Guide [33].

### 2.3. Feed and Feces Collection and Analysis

Feed samples were collected from each dietary component. Pasture grass was sampled from various points across the pasture and combined to create a global sample for analysis. Pelleted concentrates were sampled from each production batch; the resulting analytical values were averaged to determine the nutritive value of the ration. Fecal samples were collected transrectally both at the beginning and at the end of the experimental period and were analyzed individually.

Proximate analyses for feed and feces were carried out in accordance with the Official Methods of Analysis of AOAC International, 21st ed. Dry matter (DM) was determined gravimetrically by oven-drying at 105 °C to constant weight in a Binder oven (model 09-11881; AOAC 930.15, Binder GmbH, Tuttlingen, Germany), and percentage dry matter was calculated from the weight loss. Ether extract (EE) was measured by automated Soxhlet extraction with a FOSS extractor (model 2055; AOAC 920.39, FOSS Analytical AB, Hoganas, Sweden), the final fat content being expressed as percentage of the original sample weight. Crude protein (CP) was quantified by the Kjeldahl method (AOAC 984.13) using a FOSS Kjeltec digestion/distillation system (models 8400–8420, FOSS Analytical AB, Hoganas, Sweden); nitrogen values were converted to protein by the factor 6.25. Crude fibre (CF) was analyzed with a FOSS Fibertec equipment (model 2010; AOAC 978.10, FOSS Analytical AB, Hoganas, Sweden) and expressed as percentage using the standard fibre calculation. The nitrogen-free extract (NfE) was calculated by subtracting the sum of the other proximate components from 100 [34].

Digestible energy (ED) was calculated according to Martin Rosset (2021) using the equation: ED (MJ/kg) = −3.54 + (0.0209 × CP) + (0.0420 × EE) + (0.0001 × CF) + (0.0185 × NfE). Apparent digestibility (AD%) of nutrients was computed after Tassone et al. (2020): AD (%) = ((I − E)/I) × 100, where I is intake and E is excretion. The following parameters were determined: apparent dry matter digestibility (ADMD), apparent crude protein digestibility (ACPD), apparent ether extract digestibility (AEED) and apparent crude fibre digestibility (ACFD) [35,36].

### 2.4. Milk Sampling and Analyses

Individual milk samples were collected daily from each lactating jenny throughout the experimental period to monitor changes in milk composition and microbiological quality. Samples obtained on the first and last day of the experiment were specifically analyzed for both chemical composition and total bacterial count, whereas during the intermediate days, chemical composition was determined daily for each animal in all three experimental groups. Additionally, weekly milk yield was recorded individually to evaluate production dynamics over time. All results—including daily chemical composition data and microbiological measurements before and after supplementation—are presented as mean ± standard deviation (SD) for each group (n = 10). All samples were analyzed in triplicate, and the resulting mean values were used for statistical analysis. Milking was carried out manually once per day in the morning, following the temporary separation of foals. This method reflects standard practice in extensive donkey farming systems and was consistently applied throughout the experimental period.

Before sampling, the udders were cleaned, and the initial streams were discarded. Milk was then hand-milked into sterile containers, which were labelled with the collection time and animal ID. The milk was cooled and transported at approximately 4 °C, maintaining this temperature until analysis (within 24 h). The contents of the milk such as fat, protein, lactose, and total solids were analyzed using infrared spectroscopy with a Lactoscope analyser (Agro Legato International, Budapest, Hungary), in a certified lab.

For the total viable count (TVC) determination on fresh milk, samples were sent to the external laboratory AMS 2000 Trading Impex S.R.L. (in a certified lab, Jebel, Timiș County, Romania). The samples were homogenized and serially diluted in sterile peptone saline; from each dilution, 1 mL was plated onto sterile Petri dishes, overlaid with liquid nutrient agar and incubated at 37 °C for 48 h following the APHA Standard Methods for the Examination of Dairy Products (17th ed.). After incubation, colonies were counted and TVC was calculated as TVC = ΣC/(V × N × D), where ΣC is the total colony count, V the inoculated volume, N the number of plates and D the dilution factor. Results are expressed as colony-forming units per millilitre (CFU/mL). Finally, values are presented in logarithmic form (log_10_ CFU/mL) without altering the raw data [37].

### 2.5. Statistical Analysis

Statistical analyses were conducted to identify variations and significant differences among the experimental groups. For all variables—including daily milk yield, apparent digestibility parameters, and milk chemical composition—the arithmetic mean, standard deviation (SD), and coefficient of variation (CV%) were calculated. Within-group pre–post comparisons were evaluated using the paired *t*-test, whereas comparisons between groups (control, G1, G2) were assessed using the independent samples *t*-test. All analyses were performed using GraphPad Prism v. 9.0 (GraphPad Software, San Diego, CA, USA), a widely utilized platform for biomedical data analysis. Differences were deemed significant at *p* < 0.05 and highly significant at *p* < 0.001.

## 3. Results

### 3.1. Digestibility of Feed Rations

Based on the data obtained from the apparent digestibility analyses (ADMD, ACPD, AEED, ACFD) in the three experimental groups, relevant interpretations can be formulated regarding the influence of the diet on digestive efficiency in lactating jennies (Table 3).

The control group (CG), fed pasture grass and corn, showed the lowest digestibility values, particularly for crude protein (ACPD = 45.26 ± 9.85%), with high variability (CV = 21.77%), indicating poor utilization of dietary nitrogen in the absence of supplementation. In contrast, group G1, which received a diet enriched with sunflower meal and phytogenic additive, achieved the best results, recording significantly higher protein digestibility (ACPD = 57.89 ± 4.21%, *p* = 0.0028) and reduced variability (CV = 7.27%), along with elevated values for ADMD and ACFD. Group G2 showed intermediate performance, with ACPD = 55.86 ± 6.66%, significantly higher than CG (*p* = 0.012) but not different from G1 (*p* = 0.503), suggesting a primary effect of protein intake and a secondary effect of the phytogenic additives. Fat digestibility (AEED) was high and comparable between groups (≈79%, *p* > 0.05), and ACFD values were improved in the groups with enriched diets, without significant differences. The results highlight that dietary supplementation contributes to improved digestibility, particularly of protein, with G1 demonstrating the highest nutritional efficiency.

### 3.2. Milk Production Response to Feeding Strategies

**Figure 1:**
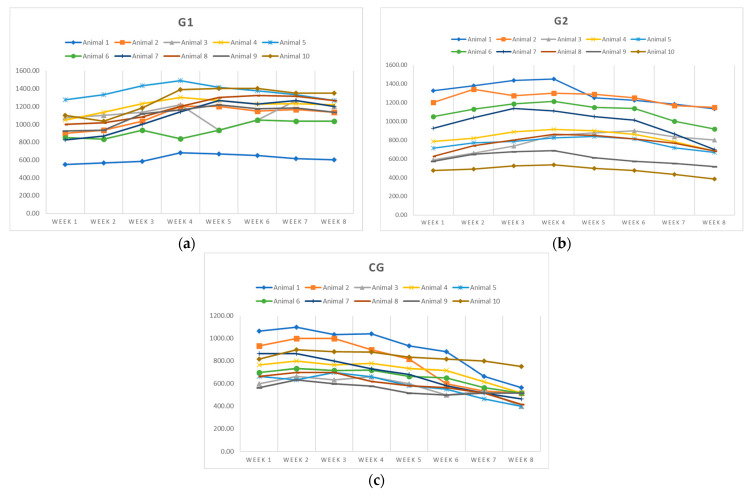
Variation in milk yield according to feeding strategies for (**a**) G1; (**b**) G2; (**c**) CG.

The evolution of milk production over the eight-week period revealed significant differences between groups, depending on the composition of the diet (Figure 1).

The control group (CG) exhibited a steady decline in milk production, from a mean of 765.00 ± 157.22 mL in week 1 to 506.67 ± 103.10 mL in week 8, with mean values significantly lower than those of the experimental groups. In contrast, group G1, receiving a diet supplemented with sunflower meal and phytogenic additive, reached a peak of 1176.67 ± 218.04 mL in week 7 and maintained high production until the end (1140.00 ± 208.79 mL). Group G2 showed a production peak in week 4 (975.00 ± 285.83 mL), followed by a decline. Individual variability was highest in G2 (CV = 34.86% in week 1) and lowest in G1. The differences between all groups were highly statistically significant (CG vs. G1: *p* = 0.000334; CG vs. G2: *p* = 0.000216; G1 vs. G2: *p* = 0.000765). These results highlight the beneficial effect of nutritional supplementation, particularly phytogenics, on sustaining and stimulating milk production in lactating jennies.

### 3.3. Dietary Influence on the Chemical Composition of Donkey Milk

The analysis of the chemical composition of donkey milk revealed significant differences among the three experimental groups, both in terms of the evolution of pre- and post-experiment values and in relation to internal variability (Table 4).

In the CG, a significant decrease in fat content (from 0.2200% to 0.1580%, *p* = 0.035) and protein (from 2.5920% to 1.6140%, *p* < 0.001) was observed, alongside a significant increase in lactose (*p* < 0.001) and a reduction in total solids (*p* = 0.002), reflecting an overall decline in milk composition in the absence of dietary intervention. Group 1 showed good stability in protein and fat content (no significant differences), but also a significant decrease in total solids (*p* = 0.011), indicating a partially favourable response. In G2, a significant decrease in protein (*p* = 0.001) was noted, along with significant increases in lactose (*p* < 0.001) and total solids (*p* < 0.001), with minimal post-experiment variability. These results suggest that dietary interventions, particularly those including phytogenic additive, can help maintain milk quality, whereas a simple diet promotes a marked compositional decline.

Statistically significant differences were observed between experimental groups in both the pre- and post-experimental phases regarding milk composition. Before supplementation, the most notable differences were found between CG and G1 for fat (*p* = 0.0001) and protein content (*p* < 0.0001), and between G1 and G2 for lactose (*p* = 0.0223) and dry matter (*p* < 0.0001). Following dietary supplementation, G1 continued to show significant differences compared to both CG and G2. The strongest differences were observed between G1 and G2 for fat (*p* = 0.0027), protein (*p* = 0.0081), and dry matter (*p* = 0.0179), and between CG and G1 for fat (*p* = 0.0003) and protein (*p* = 0.0032). Additionally, differences between CG and G2 were noted for fat (*p* = 0.011) and dry matter (*p* = 0.0021). These findings indicate that the nutritional interventions, particularly the herbal supplementation in G1, significantly influenced milk composition, leading to improved values for key components such as fat, protein, and dry matter.

### 3.4. Microbiological Quality of Donkey Milk

**Figure 2:**
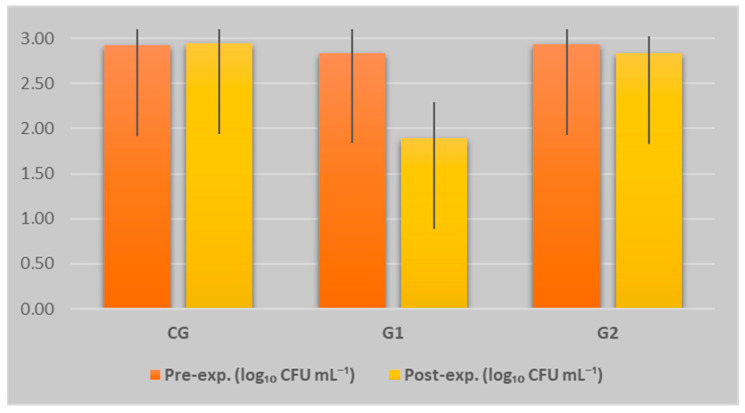
Dynamics of TVC (including SD) across experimental groups and phases. Each data point represents the mean value for 10 jennies per group (n = 10).

In the present study, the analysis of total viable counts (TVC) obtained before and after the experimental period for the three groups provided valuable information regarding the influence of the feeding protocol on milk hygiene (Figure 2). The total viable count (TVC) was expressed in logarithmic units as log CFU mL^−1^, representing the base 10 logarithm of the number of colony-forming units per millilitre of milk, in accordance with microbiological standards used to assess the hygienic quality of dairy products [37].

In G1, which received a diet supplemented with sunflower meal and phytogenic additive, a significant reduction in TVC was observed, from 2.848 ± 0.265 to 1.898 ± 0.404 (*p* = 0.000303), indicating a marked improvement in milk hygiene. Group 2 maintained relatively stable values (2.930 ± 0.260 → 2.838 ± 0.196; *p* = 0.356641), accompanied by reduced interindividual variability. In contrast, CG exhibited a slight increase in TVC (2.922 ± 0.253 → 2.949 ± 0.323; *p* = 0.792259) and greater variability, suggesting a negative trend. These findings indicate that the composition of the feed ration has a significant impact on the hygienic quality of donkey milk, both through direct effects on milk secretion and potential systemic mechanisms (e.g., immunity, digestion, absorption). Functional additives, particularly phytogenic compounds, appear to play an important role in reducing microbial load.

## 4. Discussion

A comparative analysis of the results obtained in this study provides an integrated perspective on the influence of nutritional and phytogenic strategies applied to the feeding of lactating jennies on productive performance, digestibility parameters, and milk quality. The differentiated evaluation of digestibility coefficients highlights a direct relationship between the qualitative composition of the ration and the efficiency of nutrient utilization in the organism. Complex rations, rich in plant-based protein sources and complemented with natural functional additives, promote better utilization of dry matter, protein, and fibre, thereby improving the lactogenic response and supporting the productive performance of lactating jennies. G1 clearly stands out as having the highest overall digestibility, which supports the effectiveness of a well-balanced diet not only in terms of gross nutritional value but also regarding the quality and bioavailability of the administered nutrients.

A recent study on lactating jennies found that those with medium to high crude protein levels (13.1–15.3%) had better milk production and higher apparent digestibility of dry matter and protein compared to a low-protein group [20]. This underscores the importance of optimal crude protein intake for digestion and lactation performance [20]. Additionally, another study showed that in vivo digestibility of dry matter and NDF was significantly higher than in vitro measurements, highlighting the limitations of using fecal inoculants for estimating digestive fermentation [36]. While the study did not directly examine the influence of functional plants, it stressed the importance of accurate digestibility assessment. In studies varying forage proportions, in vivo crude fibre digestibility ranged from 22 to 40%, with a meta-analysis indicating increased efficiency in donkeys due to longer fermentation in the colon and cecum [38]. Our study, showing digestibility values over 67%, supports these findings and emphasizes the benefits of a balanced diet and functional components for improving digestibility and lactation performance.

The correlation between the chemical composition of the supplement and the evolution of milk production underscores the critical importance of achieving a complex nutritional balance in the feeding of lactating jennies. It is increasingly evident that not only energy value and crude protein content are essential, but also the presence of functional compounds, such as herbal plants with galactagogue activity, which may support the hormonal and metabolic mechanisms involved in lactogenesis. Among all three groups, G1 demonstrated superior lactogenic performance, characterized by both higher milk yields and remarkable temporal stability. These findings confirm the efficacy of a well-formulated diet that combines essential nutrients with active phytogenic compounds to sustain productive performance throughout lactation.

Our results align with previous studies highlighting the importance of high-quality nutrition for sustainable lactation. For instance, Tong et al. (2024) found that selenium yeast supplementation improved lactation performance and immune response in jennies [39]. This suggests that energy solely from corn is inadequate for long-term lactation; additional bioactive components are essential for stimulating metabolic pathways. In our study, G1, which received a phytogenic additive, showed increased milk production that was sustained in later weeks, indicating the crucial role of phytogenic compounds in lactogenesis. Conversely, G2, which had the same base diet without the phytogenic additive, experienced increased milk yield until week 4, followed by a decline. This trend supports prior research, which indicates that while high-quality protein can enhance early lactation, the lack of functional factors may lead to reduced milk production later on. Although the literature on lactating jennies remains relatively scarce, recent evidence supports these conclusions. For instance, Zhou et al. (2024) conducted a study on the effects of dietary supplementation with fermented *Codonopsis pilosula* residues (FCPR) in lactating jennies, finding that it significantly increased milk production and improved blood antioxidant levels [18]. Their research supports the importance of high-quality nutritional inputs for lactation. Moreover, studies on microelement supplementation have produced mixed results. While increased mineral concentrations alone have not consistently influenced milk production or composition in jennies, evidence indicates that basic nutrient provision combined with essential amino acids can positively affect these parameters [40]. Additionally, the recent literature highlights the dynamic nature of donkey milk composition during lactation: protein levels tend to decline gradually as lactation progresses, and both biochemical and functional characteristics are influenced by the lactational stage [41]. In this context, our findings suggest that robust nutritional support—particularly diets enriched with phytogenic additives as in G1—may mitigate these natural declines more effectively than supplementation lacking functional compounds.

Overall, the correlation between dietary supplementation and milk production observed in our study aligns not only with the conclusions regarding the necessity of nutritional balance but also with findings that the inclusion of phytogenic additives can significantly enhance lactogenic performance [42,43]. Moreover, the natural dynamics of lactation, characterized by declining milk production and protein content over time, are well-documented in the literature and corroborated by our data. Consequently, the present study contributes to the scientific body of knowledge by highlighting the synergistic effect of combined protein, energy, and phytogenic inputs on the sustainable milk production of lactating jennies [31,32]. In the context of recently published research, complex nutritional support incorporating functional compounds appears to be key for optimizing lactation in donkey farms.

Scientific evidence further supports that the addition of bioactive components like herbal supplements or functional nutrients, directly influences the structure of the gut microbiota, leading to improved fermentation, enhanced digestibility of protein, fibre, and dry matter, and, consequently, greater nutritional and lactational efficiency. Our findings align with this paradigm: the increase in digestibility coefficients in the group supplemented with phytogenic additives may be explained by the positive modulation of the hindgut microbiota, facilitating fermentation and efficient nutrient absorption. This integrative relationship between ration composition, gut microbiota, digestibility, and lactogenic performance is further supported by the cited studies, providing a scientific foundation and practical applications for optimizing the nutrition of lactating jennies [44,45,46].

Within the framework of the present study, the assessment of the hygienic quality of donkey milk represented a central objective, given its direct implications for nutritional value and consumer safety. Raw donkey milk typically exhibits low bacterial loads when appropriate hygiene practices are applied, with TVC values generally ranging from 2.4 to 5.9 log_10_ CFU mL^−1^, as reported in several studies [6,47,48,49,50,51,52,53,54]. In our study, the pre-intervention TVC values for all three groups (G1, G2, and CG) fell within this reported range, indicating a generally good baseline hygiene status across the herd. Similar observations have been reported in a study of donkey milk from two Greek breeds, where TVC values ranged between 2.18 and 2.71 log_10_ CFU mL^−1^. In that case, animals were milked manually in a traditional setting, and the bacterial load remained low [5], closely corresponding to the initial values recorded in the present study. Furthermore, another investigation analyzing bacterial contamination in donkey milk from five farms employing different milking methods (manual and mechanical) reported a mean TVC of 5.38 log_10_ CFU mL^−1^. The lowest value, 2.84 log_10_ CFU mL^−1^, was observed in an extensive grazing system where animals had free access to pasture, and milking was performed manually and irregularly [48]. These findings align with the microbiological reference values obtained in G1 of the current study. Following the dietary intervention, only G1, which received the phytogenic additives, demonstrated a significant reduction in TVC, whereas G2 and CG remained largely unchanged. These results support the antimicrobial efficacy of herbal feed additives in donkey milk production. Collectively, the findings suggest a promising, natural strategy for enhancing milk hygiene, warranting further investigation into specific bioactive interactions and their potential applications in sustainable dairy systems.

The nutritional quality of donkey milk, in terms of its constituent components, is well-documented in the literature and is characterized by low fat and protein content but high lactose levels. In the present study, the differences observed among the three experimental groups were clearly influenced by the applied nutritional protocol. Across all groups, a tendency toward reduced fat content was noted following the experimental phase; however, this decrease was less pronounced in the group receiving phytogenic supplements, suggesting a potential protective effect of these additives on lipid metabolism. This observation is supported by the findings of Malacarne et al. (2019), who emphasized that the stage of lactation influences the lipid composition of donkey milk, with values below 0.5% considered typical for this species [55]. Regarding protein content, the most pronounced changes were associated with the CG, whereas the experimental groups exhibited a smaller decline, particularly in G1. Donkey milk typically contains between 1.5% and 2.0% protein, as reported by Martini et al. (2018), and while dietary inputs can influence the synthesis of milk proteins, such effects are generally moderate [1]. The increase in lactose content observed in all groups at the end of the experiment may be correlated with metabolic changes induced by the applied feeding protocols. Elevated lactose levels, ranging between 5.8% and 7.4%, are characteristic of donkey milk and are associated with good digestibility and compositional similarity to human milk [5,55,56]. These observations suggest a relationship between nutritional intake and the synthesis of milk carbohydrates. As for total solids, this parameter remained stable in the experimental groups, while a slight decrease was noted in CG. According to the literature, normal values for total solids in donkey milk range between 8.5% and 9.8% [48,50], consistent with the range observed in the present study. Overall, the nutritional intervention based on phytogenic additives exerted a stabilizing effect on the chemical composition of the milk, mitigating variations associated with advancing lactation stages. This effect may be attributed to the action of plant-derived compounds with galactagogue, immunomodulatory, and antioxidant properties, which support mammary gland function and help maintain consistent milk quality throughout lactation [21].

In conclusion, the data obtained in this study align with trends reported in the literature and demonstrate that carefully formulated nutritional interventions can have beneficial effects on digestibility and milk quality in lactating jennies. The positive impact is particularly evident when the diet is balanced in terms of energy and protein and complemented with functional additives of plant origin. These findings may serve as a foundation for developing optimized feeding strategies with practical applications aimed at improving productive performance and product quality in donkey farming systems.

The strengths of this study include its comparative design across three groups, implementation under real farm conditions, integrated evaluation of milk production, chemical composition, and microbial load, standardized administration of the phytogenic additives blend in pellet form, individual sample collection, and the maintenance of milk parameters within species-specific ranges without evident adverse effects.

The limitations of the study are the relatively small sample size (10 animals per group), which restricts the power to detect moderate effects; its conduct within a single farm, limiting the generalizability of findings to other systems, breeds, or microclimatic conditions; and the limited duration (8 weeks), which is insufficient to assess the persistence of effects over the entire lactation cycle. In addition, the economic feasibility of supplement inclusion was not assessed, and future research should consider targeted cost–benefit analyses, particularly for small-scale production systems.

Future research should aim to test the phytogenic additives under different management systems, monitor the entire lactation curve and weaning period, include a post-supplementation phase to evaluate the persistence of effects, and employ sequencing methods to characterize changes in the milk and fecal microbiome. Incorporating measurements of lysozyme, lactoferrin, and inflammatory/oxidative markers would help clarify the underlying mechanisms. Overall, phytonutritional supplementation emerges as a promising strategy to enhance both the quantity and quality of donkey milk, opening concrete perspectives for the sustainable expansion of this sector.

## 5. Conclusions

In lactating jennies raised under extensive conditions, pasture-based diets supplemented with cereals, protein meals, and functional additives increased milk yield, maintained milk composition, and reduced microbial loads in raw milk. The phytogenic supplement with galactagogue activity produced the most favourable outcomes, improving milk output, nutrient digestibility, and udder health. These results highlight the potential of phytogenic feed additives as natural tools to enhance productivity and hygiene in sustainable donkey-milk production systems.

## Figures and Tables

**Table 1:** Dietary formulation and nutrient composition.

		CG	G1	G2
		**Ingredients**		
**Pasture grass** **consumed**	**kg**	**11**		
		**Chemical composition (% as is)**		
DE	MJ/kg	4.13		
DM	%	60.60		
CP	%	11.80		
EE	%	3.80		
CF	%	27.50		
NfE	%	9.6		
		**Ingredients**		
**Compound feed consumed (% as is)**	**kg**	**1**	**1**	**1**
Corn (ground)	%	100.00	36.00	39.00
Wheat (ground)	%	0.00	53.60	53.60
Sunflower meal	%	0.00	7.00	7.00
Herbal supplement	%	0.00	3.00	0.00
Bentonite	%	0.00	0.40	0.40
Total	%	100.00	100.00	100.00
		**Chemical composition (% as is)**		
DE	MJ/kg	5.13	5.14	5.12
DM	%	85.68	89.43	86.91
CP	%	14.08	22.37	20.98
EE	%	2.46	1.89	2.08
CF	%	2.46	5.22	4.67
NfE	%	64.78	57.43	56.82

DE = digestible energy Mj/kg; DM = dry matter; CP = crude protein; EE = ether extract; CF = crude fibre; NfE = nitrogen-free extract.

**Table 2:** Formulation of the phytogenic additive.

Name	Quantity/kg (As Is)
Lemon balm (*Melissa officinalis*)	179.2 g
Dill (*Anethum graveolens*)	179.2 g
Fennel (*Foeniculum vulgare*)	92.8 g
Blessed thistle (*Cnicus Benedictus*)	34.6 g
Raspberry (*Rubus idaeus*)	92.8 g
Hop (*Humulus lupulus*)	34.6 g
Elders flower (*Sambucus*)	34.6 g
Stinging Nettle (*Urtica dioica*)	76.5 g
Chamomile (*Matricaria chamomilla*)	179.2 g
Fenugreek (*Trigonella foenumgraecum*)	50.7 g
Turmeric (*Curcuma longa*)	9.4 g
Ginger (*Zingiber officinale*)	15.7 g
Anise (*Pimpinella anisum*)	20.7 g
**Chemical composition (% as is)**	
DM	91.74%
CP	18.74%
EE	1.4%
CF	35.63%

DM = dry matter; CP = crude protein; EE = ether extract; CF = crude fibre.

**Table 3:** Apparent digestibility of tested diets for donkeys.

Group	Parameter	Mean	Min.–Max.	x ± SD	CV (%)	*p*-Value
**CG**	ADMD	58.72	47.38–72.35	58.72 ± 6.74	11.47	0.1314
	ACPD	45.26	19.58–56.71	45.26 ± 9.85	21.77	0.5030
	AEED	78.27	71.77–85.36	78.27 ± 3.70	4.73	0.4295
	ACFD	67.65	53.21–77.91	67.65 ± 6.92	10.22	0.2275
**G1**	ADMD	62.82	55.40–72.41	62.82 ± 4.56	7.26	0.4874
	ACPD	57.89	48.63–62.10	57.89 ± 4.21	7.27	0.0028
	AEED	79.3	76.14–82.15	79.30 ± 2.36	2.97	0.4692
	ACFD	71.19	61.77–76.63	71.19 ± 5.70	8.01	0.3044
**G2**	ADMD	60.62	52.00–69.37	60.62 ± 5.13	8.46	0.3261
	ACPD	55.86	48.95–72.60	55.86 ± 6.66	11.91	0.0123
	AEED	79.28	72.02–83.66	79.28 ± 3.35	4.23	0.9891
	ACFD	70.64	57.53–75.65	70.64 ± 5.69	8.05	0.8315

ADMD = apparent dry matter digestibility; ACPD = apparent crude protein digestibility; AEED = apparent ether extract digestibility; ACFD = apparent crude fibre digestibility.

**Table 4:** Effect of diet on the chemical composition of donkey milk.

Group	Parameter %	Pre-Experimental				Post-Experimental				*p*-Value
		Mean	Min–Max	*x* ± SD	CV (%)	Mean	Min–Max	*x* ± SD	CV (%)	
**CG**	Fat	0.22	0.18–0.26	0.2200 ± 0.0258	11.74	0.158	0.05–0.22	0.1580 ± 0.0581	36.76	0.035131
	Protein	2.592	2.13–2.84	2.5920 ± 0.2219	8.56	1.614	1.37–1.91	1.6140 ± 0.1559	9.66	0.000002
	Lactose	6.005	5.89–6.12	6.0050 ± 0.0717	1.19	6.385	6.11–6.69	6.3850 ± 0.2052	3.21	0.000059
	Total solids	9.513	8.93–9.89	9.5130 ± 0.3511	3.69	8.873	8.51–9.15	8.8730 ± 0.2012	2.27	0.002438
**G1**	Fat	0.27	0.25–0.29	0.2700 ± 0.0141	5.24	0.258	0.22–0.3	0.2580 ± 0.0312	12.09	0.205528
	Protein	1.91	1.68–2.08	1.9100 ± 0.1234	6.46	1.817	1.68–1.94	1.8170 ± 0.0952	5.24	0.074022
	Lactose	6.307	5.79–6.67	6.3070 ± 0.3156	5.0	6.475	6.11–6.74	6.4750 ± 0.2017	3.11	0.127845
	Total solids	9.299	9.02–9.66	9.2990 ± 0.2186	2.35	9.006	8.84–9.26	9.0060 ± 0.1419	1.58	0.010864
**G2**	Fat	0.257	0.16–0.4	0.2570 ± 0.0718	27.94	0.217	0.19–0.24	0.2170 ± 0.0177	8.14	0.097778
	Protein	1.923	1.57–2.11	1.9230 ± 0.2049	10.65	1.67	1.52–1.85	1.6700 ± 0.1223	7.32	0.001454
	Lactose	6.012	5.75–6.39	6.0120 ± 0.1820	3.03	6.476	6.33–6.61	6.4760 ± 0.0947	1.46	0.000135
	Total solids	8.866	8.71–9.06	8.8660 ± 0.1300	1.47	9.141	9.02–9.22	9.1410 ± 0.0687	0.75	0.000163

## Data Availability

The data supporting the findings of the study are available within the article and Supplementary file.

## Supplementary Materials

The following supporting information can be downloaded at https://www.mdpi.com/article/10.3390/ani15203060/s1: Table S1. Milk Production in Donkey Groups During the Experimental Trial.
