# Supplementary material for: Phytogenic and Nutritional Strategies to Improve Milk Production and Microbiological Quality in Lactating Donkeys
Source: Animals (Basel). 2025 Oct 21;15(20):3060. doi: 10.3390/ani15203060 (PMC12561713; doi:10.3390/ani15203060)
Supplement: Supplementary file 1 [file animals-15-03060-s001.zip › animals-3895553-supplementary.pdf]

Table S1. Milk Production in Donkey Groups During the Experimental Trial

Group	Week	Mean	Min.-Max.	X ± SD	CV (%)
CG	WEEK 1	765.0	566.67-1066.67	765.00 ± 157.22	20.55
	WEEK 2	803.33	633.33-1100	803.33 ± 160.59	19.99
	WEEK 3	783.33	600.0-1033.33	783.33 ± 146.99	18.76
	WEEK 4	757.0	580.0-1040.0	757.00 ± 144.23	19.05
	WEEK 5	695.0	516.67-933.33	695.00 ± 132.88	19.12
	WEEK 6	636.67	500.0-883.33	636.67 ± 130.72	20.53
	WEEK 7	573.34	466.67-800.0	573.34 ± 97.88	17.07
	WEEK 8	506.67	400.0-750.0	506.67 ± 103.10	20.35
G1	WEEK 1	955.0	550.0-1275.0	955.00 ± 195.72	20.49
	WEEK 2	975.0	566.67-1333.33	975.00 ± 204.46	20.97
	WEEK 3	1073.33	583.33-1433.33	1073.33 ± 221.00	20.59
	WEEK 4	1162.0	680.0-1490.0	1162.00 ± 240.68	20.71
	WEEK 5	1160.0	666.67-1416.67	1160.00 ± 239.62	20.66
	WEEK 6	1162.5	650.0-1400.0	1162.50 ± 217.07	18.67
	WEEK 7	1176.67	616.67-1350.0	1176.67 ± 218.04	18.53
	WEEK 8	1140.0	600.0-1350.0	1140.00 ± 208.79	18.31
G2	WEEK 1	826.25	475.0-1325.0	826.25 ± 288.04	34.86
	WEEK 2	902.0	490.0-1380.0	902.00 ± 304.33	33.74
	WEEK 3	945.0	525.0-1437.5	945.00 ± 296.05	31.33
	WEEK 4	975.0	537.5-1450.0	975.00 ± 285.83	29.32
	WEEK 5	931.25	500.0-1287.5	931.25 ± 257.68	27.67
	WEEK 6	906.25	475.0-1250.0	906.25 ± 258.15	28.49
	WEEK 7	830.0	433.33-1183.33	830.00 ± 241.11	29.05
	WEEK 8	763.33	383.33-1150.0	763.33 ± 245.55	32.17
